# Ischemia-Like Stress Conditions Stimulate Trophic Activities of Adipose-Derived Stromal/Stem Cells

**DOI:** 10.3390/cells9091935

**Published:** 2020-08-21

**Authors:** Julia Bachmann, Elias Ehlert, Matthias Becker, Christoph Otto, Katrin Radeloff, Torsten Blunk, Petra Bauer-Kreisel

**Affiliations:** 1 Department of Trauma, Hand, Plastic and Reconstructive Surgery, University of Wuerzburg, 97080 Wuerzburg, Germany; Bachmann_J1@ukw.de (J.B.); Ehlert_E@ukw.de (E.E.); Blunk_T@ukw.de (T.B.); 2 Institute for Medical Radiation and Cell Research, University of Wuerzburg, 97078 Wuerzburg, Germany; Matthias.Becker@uni-wuerzburg.de; 3 Department of General, Visceral, Transplantation, Vascular and Pediatric Surgery, University of Wuerzburg, 97080 Wuerzburg, Germany; Otto_C@ukw.de; 4 Department of Otorhinolaryngology, Head and Neck Surgery, Carl von Ossietzky-University of Oldenburg, 26133 Oldenburg, Germany; Katrin.Radeloff@uni-oldenburg.de

**Keywords:** adipose-derived stromal/stem cells (ASCs), regenerative medicine, secretion, trophic factors, ischemia, glucose starvation, hypoxia

## Abstract

Adipose-derived stromal/stem cells (ASCs) have been shown to exert regenerative functions, which are mainly attributed to the secretion of trophic factors. Upon transplantation, ASCs are facing an ischemic environment characterized by oxygen and nutrient deprivation. However, current knowledge on the secretion capacity of ASCs under such conditions is limited. Thus, the present study focused on the secretory function of ASCs under glucose and oxygen deprivation as major components of ischemia. After exposure to glucose/oxygen deprivation, ASCs maintained distinct viability, but the metabolic activity was greatly reduced by glucose limitation. ASCs were able to secrete a broad panel of factors under glucose/oxygen deprivation as revealed by a cytokine antibody array. Quantification of selected factors by ELISA demonstrated that glucose deprivation in combination with hypoxia led to markedly higher secretion levels of the angiogenic and anti-apoptotic factors IL-6, VEGF, and stanniocalcin-1 as compared to the hypoxic condition alone. A conditioned medium of glucose/oxygen-deprived ASCs promoted the viability and tube formation of endothelial cells, and the proliferation and migration of fibroblasts. These findings indicate that ASCs are stimulated by ischemia-like stress conditions to secrete trophic factors and would be able to exert their beneficial function in an ischemic environment.

## 1. Introduction

Adipose tissue-derived stromal/stem cells (ASCs) represent a valuable tool for cell-based therapies because of their widely acknowledged capacity to exert beneficial functions in tissue regeneration or in tissue repair [1,2,3,4,5,6]. This has been demonstrated for example for cell-assisted lipotransfer, where autologous ASCs added to lipografts have been shown to enhance vascularity, to improve the survival rate of grafts, and to reduce postoperative atrophy [7,8,9]. The benefits of ASCs in this context were mainly attributed to secreted paracrine factors rather than to direct differentiation into tissue-specific cell types [10,11]. In recent years, numerous studies have shown that ASCs secrete a complex panel of trophic factors, including growth factors, cytokines, chemokines, extracellular microvesicles and exosomes, that contribute to angiogenesis, anti-apoptosis, immunomodulation, and the activation of resident and circulating stem cells [10,11,12,13,14,15,16,17]. However, in regenerative approaches such as tissue engineering or cell-assisted lipotransfer, the implanted cell-laden construct or lipograft is at least initially impaired by a lack of blood supply [18,19]. This leads to an ischemic environment that is characterized by the deprivation of nutrients, oxygen, and growth factors. It has been shown that depletion of oxygen and nutrients, in particular glucose, significantly affects cell survival and function, as they are both critically required for energy-related pathways [20,21,22].

To date, the response of ASCs to ischemic stress and the different components of ischemia remains poorly understood. Only a few studies so far examined the viability and metabolic response of ASCs to combined oxygen and glucose deprivation, but they did not focus on their secretory function under this condition [23,24]. The modulation of the paracrine activity of ASCs by low oxygen concentrations is well documented. However, little is known about how nutrient deprivation, a further major component of ischemic stress, can affect their secretory potential.

In this context, the present study aimed to investigate the effect of glucose and oxygen deprivation on the viability, metabolic activity, and secretory capacity of ASCs. Specifically, we focused on glucose starvation in concert with hypoxia as a potential modulator of the paracrine function of ASCs. In response to combined glucose and oxygen deprivation, ASCs demonstrated increased levels of secreted angiogenic and anti-apoptotic mediators including vascular endothelial growth factor (VEGF), interleukin-6 (IL-6), IL-8, angiogenin (ANG), and stanniocalcin-1 (STC-1). We further investigated the impact of conditioned medium of ischemia-challenged ASCs on the viability and tube formation of endothelial cells, and the proliferation and migration of fibroblasts. The results of this study suggest that ASCs can maintain their secretory function and thus exert regenerative effects even under ischemia-like stress conditions.

## 2. Materials and Methods

### 2.1. Cells and Cell Culture

Human adipose-derived stem cells (ASCs) were obtained from Lonza (Walkersville, MD, USA). ASCs were expanded and cultured in 175 cm^2^ cell culture-treated plastic flasks in growth medium consisting of Dulbecco’s Modified Eagle´s Medium/Ham´s F-12 (DMEM/F-12) (Thermo Scientific, Waltham, MA, USA), supplemented with 1% penicillin/streptomycin (Thermo Scientific), 10% fetal bovine serum (FBS; Thermo Scientific), and 3 ng/mL basic fibroblast growth factor (bFGF; BioLegend, London, UK) dissolved in phosphate-buffered saline (PBS) containing 1% BSA (bovine serum albumin). Cultures were maintained under a sterile humidified 37 °C, 5% CO_2_, and 95% air environment. The culture medium was replaced every other day. At 80–85% confluence, the ASCs were detached using a 0.25% trypsin-EDTA solution (Thermo Scientific) and passaged. ASCs were used at passage 4 for the subsequent experiments. Human umbilical vein endothelial cells (HUVECs) were obtained from PromoCell (Heidelberg, Germany). HUVECs were plated in 25 cm^2^ flasks and cultured in endothelial cell growth medium 2 (PromoCell). When the cells reached 70–80% confluence, they were detached with DetachKit (PromoCell) and expanded to passages 2–3. NIH/3T3 fibroblasts were obtained from ATCC (Manassas, VA, USA). Fibroblasts were cultured in fibroblast growth medium (Dulbecco´s Modified Eagle´s Medium (Thermo Scientific), supplemented with 1% penicillin/streptomycin (Thermo Scientific), and 10% bovine calf serum (BCS; Sigma-Aldrich, Munich, Germany)). Cultures were maintained under a sterile humidified 37 °C, 5% CO_2_, and 95% air environment. The culture medium was replaced every other day. At 70–80% confluence, the fibroblasts were detached using a 0.25% trypsin-EDTA solution (Thermo Scientific) and passaged.

### 2.2. Ischemic Culture Conditions

ASCs were exposed to glucose and oxygen deprivation separately and in combination. Cells were seeded (25 × 10^3^ cells/cm^2^) in growth medium, and after incubation overnight with 21% O_2_ for attachment of cells, ASCs were washed twice with PBS and then cultured in basal medium (d-glucose-, L-glutamine-, phenol red-, and sodium pyruvate-free DMEM) containing no serum. The medium was supplemented with D-glucose (0.1 or 1 g/L) according to the respective condition. The following conditions were examined: (1) 1 g/L glucose and normoxia (21% O_2_) (control); (2) 1 g/L glucose and hypoxia (0.2% O_2_); (3) 0.1 g/L glucose and normoxia (21% O_2_); (4) 0.1 g/L glucose and hypoxia (0.2% O_2_). Hypoxic conditions were achieved using the well-established and finely controlled proOx-C chamber system (C-Chamber, C-274; BioSpherix, New York, NY, USA). The oxygen concentration was maintained at 0.2% with the residual gas mixture composed of 5% CO_2_ and balance nitrogen. In order to ensure sustained hypoxic conditions, cell cultures were left undisturbed without medium changes.

### 2.3. Live/Dead Staining

ASCs and HUVECs were seeded in their respective growth medium and after attachment of cells, they were cultured according to the respective condition. Cell viability was determined using live/dead cell staining (PromoKine, Heidelberg, Germany) according to the manufacturer´s instructions. Living cells were stained with calcein (green) and dead cells were stained with ethidium bromide (red). Images were taken using an Olympus IX51 fluorescence microscope and analyzed with the Olympus CellSens^™^ Software v1.16 (Olympus, Hamburg, Germany).

### 2.4. Quantification of DNA

For determination of total DNA content, the intercalating dye Hoechst 33,258 was used (Polysciences, Warrington, PA, USA). Cells were harvested in phosphate-buffered saline (PBS) and sonicated with an ultrasonic homogenizer. Quantification of DNA content was carried out by measuring fluorescence intensities at an excitation wavelength of 365 nm and an emission wavelength of 458 nm with a fluorescence spectrometer (Infinite M200; Tecan, Crailsheim, Germany).

### 2.5. MTT Assay

ASCs, HUVECs, and NIH/3T3 fibroblasts were seeded in their respective growth medium and after attachment of cells, they were cultured according to the respective condition. At the indicated time points, cells were treated with MTT (3-(4,5-dimethylthiazol-2-yl)-2,5-diphenyltetrazolium bromide) at a final concentration of 0.5 mg/mL and incubated for 3 h at 37 °C. Cells were then washed with phosphate-buffered saline (PBS), incubated with dimethyl sulfoxide (DMSO) for 5 min with gentle shaking, and mixed to ensure complete solubilization of the dye formed. The respective light absorbance of this solution was recorded using a microplate reader (Infinite M200; Tecan) at a wavelength of 570 nm. The mean value of day 0 samples was taken as reference and set as 100%.

### 2.6. Adipogenic Differentiation of Ischemia-Treated ASCs

To assess the adipogenic differentiation capability of ischemia-treated ASCs, cells were cultured under combined glucose/oxygen deprivation for four days. The cells were then trypsinized, re-seeded, and cultured in Preadipocyte basal medium-2 (PBM-2; Lonza) containing 10% FCS and 1% penicillin/streptomycin. After two days, adipogenic differentiation was induced by changing to differentiation medium (PBM-2 with 1.7 μM insulin (PromoCell, Heidelberg, Germany), 1 μM dexamethasone, 200 μM indomethacin (both from Sigma-Aldrich), and 500 μM 3-isobutyl-1-methylxanthin (IBMX; Serva Electrophoresis, Heidelberg, Germany)) for 14 days. Non-induced cells were cultured in PBM-2 during the differentiation period. In parallel, control ASCs were cultured in growth medium for four days and then treated in the same way for adipogenic differentiation. After 14 days of culture in adipogenic medium, lipid accumulation was histologically assessed by staining with Oil Red O solution (3 mg/mL Oil Red O in 60% isopropanol; Sigma-Aldrich) and cell nuclei were counterstained with hematoxylin (Bio Optica, Milan, Italy). Samples were imaged using an Olympus IX51 inverted light microscope and analyzed with the Olympus CellSens^™^ Software v1.16 (Olympus, Hamburg, Germany).

The quantitative determination of intracellular lipid accumulation was performed using the Serum Triglyceride Determination Kit from Sigma-Aldrich. Cells were harvested and sonified in Thesit solution (0.5% Thesit in H_2_O; Gepepharm, Hennef, Germany) and the triglyceride quantification was performed according to the manufacturer’s instructions and measured with a microplate reader (Infinite M200; Tecan) at a wavelength of 570 nm. Triglyceride contents were normalized to the DNA content of the respective samples.

### 2.7. Glucose and Lactate Determination

Exogenous glucose and lactate levels in the cell culture supernatants were measured with GLUC3 and LACT2 COBAS INTEGRA substrate reagents using the related COBAS INTEGRA 800 (Roche, Basel, Switzerland) robot.

### 2.8. Assays of Cytokines

Antibody array: To identify factors secreted by glucose/oxygen-deprived ASCs, a profiling of human cytokines was performed using an antibody array covering 80 cytokines (Human Cytokine Antibody Array C5; RayBiotech, Norcross, GA, USA). Cell culture supernatants from glucose/oxygen-deprived ASCs (0.1 g/L glucose, 0.2% O_2_) and ASCs cultured under the control condition (1 g/L glucose, 21% O_2_) were centrifuged at 1000× *g* for 5 min to remove cell debris. Array analyses were performed according to the manufacturer’s instructions. Briefly, the array membranes were blocked with a blocking buffer and incubated with 1 mL of each supernatant overnight at 4 °C. Subsequently, the membranes were assayed for chemiluminescence signals.

Enzyme-linked immunosorbent assays (ELISAs): The concentrations of individual cytokines in the cell culture supernatants from cells cultured under the different deprivation conditions and the control condition were determined using ELISA kits for vascular endothelial growth factor (VEGF), interleukin (IL)-6, IL-8, angiogenin (ANG), TIMP metallopeptidase inhibitor (TIMP)-1, monocyte chemoattractant protein (MCP)-1, and stanniocalcin (STC)-1 from R&D Systems (DuoSet ELISA; Minneapolis, MN, USA). Concentration levels were normalized to the total DNA content of the respective samples (pg/µg DNA).

### 2.9. RNA Isolation and Quantitative Real-Time PCR (qRT-PCR) Analysis

Total RNA from cultured cells was isolated using TRIzol^®^ reagent (Invitrogen, Karlsruhe, Germany). First-strand cDNA was synthesized from total RNA with ImProm-II Reverse Transcription System (Promega, Mannheim, Germany). Quantitative PCR analyses were performed using the MESA GREEN qPCR MasterMix Plus with MeteorTaq polymerase (Eurogentec, Seraing, Belgium). cDNA for genes of interest was amplified with the PrimePCR^™^ SYBR^®^ Green Assay using the following cycle conditions: 95 °C for 15 min initial denaturation followed by 40 cycles at 95 °C for 15 s, 60 °C for 30 s, and 70 °C for 30 s using the following primers: IL-6 (qHsaCID0020314, IL6, human), VEGF (qHsaCED0043454, VEGFA, human), and STC-1 (qHsaCID0006115, STC1, human), all from BioRad (Hercules, CA, USA). mRNA expression levels were normalized to the eukaryotic translation elongation factor 1 alpha (EF1α) (forward, 5′-ccccgacacagtagcatttg-3′; reverse, 5′-tgactttccatcccttgaacc-3′) (Biomers, Ulm, Germany). The relative expression levels were determined using the 2^−ΔΔCT^ method and were further normalized to the respective day 0 sample.

### 2.10. Preparation of Conditioned Medium

ASCs were seeded at 25,000 cells per cm^2^ in growth medium and allowed to adhere overnight at 21% O_2_. ASCs were washed twice with PBS, and the medium was replaced with basal medium (D-glucose-, L-glutamine-, phenol red-, and sodium pyruvate-free DMEM) containing no serum and supplemented with 0.1 g/L glucose. Cells were incubated under 0.2% O_2_, to generate a conditioned medium (CM) of ASCs exposed to glucose/oxygen deprivation. After four days, the medium was harvested as ASC-CM_ischemic_.

### 2.11. Tube Formation Assay

Angiogenesis µ-Slides (Ibidi, Gräfelfing, Germany) were coated with 10 µL of growth factor- reduced matrigel (BD Biosciences, San Jose, CA, USA). HUVECs were suspended in basal medium, ASC-CM_ischemic_ or endothelial growth medium and plated with 1 × 10^4^ cells per well on top of the matrigel. After 4, 6, and 10 h of incubation at 37 °C under hypoxic conditions (0.2% O_2_), the formation of tube-like structures was examined microscopically. The tube length and branch count were quantified using the automated image analyzer ACAS from ibidi (Tube formation ACAS image analysis module) at the indicated time points.

### 2.12. Proliferation and Metabolic Activity of Fibroblasts

The conditioned medium from glucose/oxygen-deprived ASCs (ASC-CM_ischemic_) was prepared as described. Fibroblasts were treated with basal medium (DMEM, w/o FBS) or ASC-CM_ischemic_, each supplemented with 1 g/L glucose, under normoxic conditions. Proliferation and metabolic activity of the cells were analyzed at the indicated time points using a DNA and MTT assay as described above.

### 2.13. Fibroblast Migration Assay

The migratory activity of NIH/3T3 fibroblasts was assessed using a migration assay. Ibidi Culture-Inserts 2 well (Ibidi, Gräfelfing, Germany) were transferred into 6-well plates and 70 µL cell suspension containing 3 × 10^5^ cells/mL was applied to each well. After an appropriate duration for cell attachment (24 h) the Ibidi Culture-Inserts were removed to create a cell-free gap of 500 µm. Cells were then washed with phosphate-buffered saline (PBS), and incubated with basal medium (DMEM, w/o FBS) or ASC-CM_ischemic_, each supplemented with 1 g/L glucose, under normoxic conditions for 24 h. The fibroblast growth medium was used as positive control. To monitor the progress of gap closure, micrographs were taken at different time points.

### 2.14. Statistical Analysis

Quantitative results are presented as means ± SD. Statistical analyses of variance comparisons between groups were performed using the ANOVA-test in conjunction with Bonferroni post-hoc adjustment. For statistical analyses of endothelial tube formation an unpaired Student’s *t*-test was applied. For all analyses, differences at *p* < 0.05 were considered as statistically significant. All statistical analyses were performed using the GraphPad Prism Software 8.3 (GraphPad Software, San Diego, CA, USA).

## 3. Results

### 3.1. Characterization of ASCs under Glucose/Oxygen Deprivation

Viability and metabolic activity of ASCs might be strongly dependent on the availability of glucose and the presence of oxygen. The impact of glucose and oxygen deprivation, two conditions that are expected to occur in an ischemic situation, were investigated individually and in concert. For this purpose, the cells were exposed to culture conditions represented by glucose starvation (0.1 g/L glucose) and hypoxia (0.2% O_2_) under serum-free conditions for a period of seven days.

**Figure 1:**
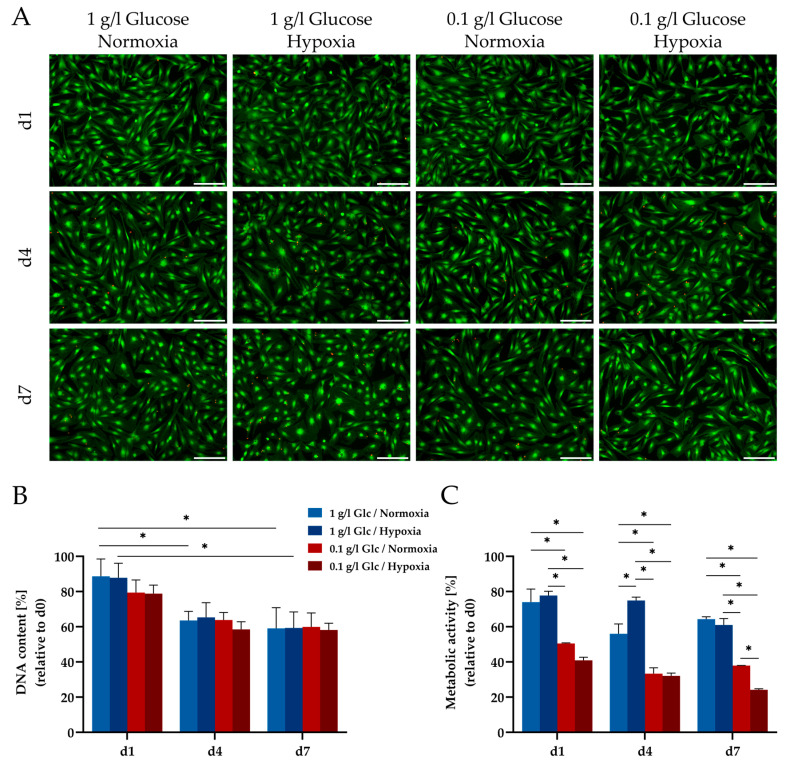
Viability and metabolic activity of human adipose-derived stromal/stem cells (ASCs) under glucose and oxygen deprivation. (**A**) Microscopic images of live/dead staining. Twenty five thousand cells per cm^2^ were seeded in well plates and imaged at the indicated time points. Living cells were stained with calcein (green) and dead cells were stained with ethidium bromide (red). Scale bar represents 200 μm. (**B**) Quantitative determination of total DNA content in relation to the mean value of day 0. (**C**) Metabolic activity as determined by a MTT assay. MTT accumulated in ASCs was solubilized and the optical density was measured at 570 nm; % metabolic activity was calculated in relation to the mean value of day 0. Data are presented as means ± SD of *n* = 3; * *p* < 0.05. Abbreviation: MTT: 3-(4,5-dimethylthiazol-2-yl)-2,5-diphenyltetrazolium bromide.

Viability of the cells was examined by live/dead staining. Under glucose deprivation or hypoxia, a similar viability was observed as in the control group (1 g/L glucose, 21% O_2_, without serum) with cells retaining their typical stellate morphology (Figure 1A). Even under combined glucose and oxygen deprivation, a considerable number of viable cells were still present after seven days. Dead (red) cells were hardly detectable in this set-up, as they rapidly detached from the cell-culture plates and consequently were washed out during the staining process. This observation was corroborated by the assessment of the cell numbers as reflected by the DNA content (Figure 1B). Deprivation conditions demonstrated comparable effects on cell numbers without significant differences between the investigated culture conditions. Across all tested conditions the DNA content decreased to approx. 60% by day 4, likely due to the lack of serum in the medium and remained stable until day 7. In contrast, the metabolic activity of the cells (determined using the MTT and the WST-8 assay) was significantly impaired by the limitation of glucose (0.1 g/L) already at day 1 and also at day 4, independent of the oxygen concentration (Figure 1C and Figure S1).

**Figure 2:**
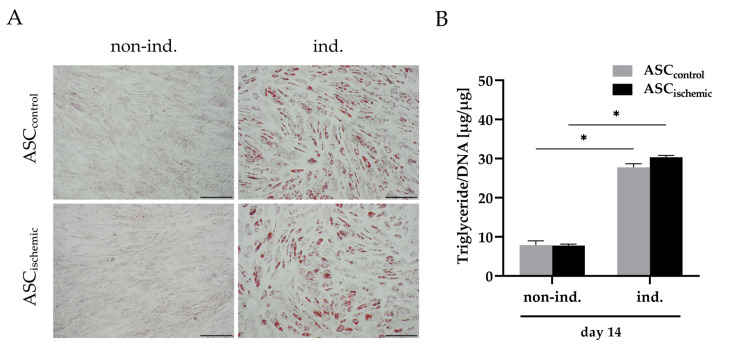
Adipogenic differentiation potential of ASCs after exposure to ischemia-like conditions. ASC_ischemic_ were cultured under glucose/oxygen deprivation (0.1 g/L glucose, 0.2% O_2_) for four days before being adipogenically induced. ASC_control_ were cultured in growth medium before induction. (**A**) Histological analysis of adipogenesis after 14 days of induction by staining with Oil Red O (ORO, red) and hematoxylin (blue). Non-induced cells served as controls. Representative images are shown. Scale bar represents 200 μm. (**B**) Quantitative analysis of triglyceride content. Analysis was performed at day 14. Triglyceride contents were normalized to the DNA content of the respective samples. Data are presented as means ± SD of *n* = 3; * *p* < 0.05. Abbreviation: Non-ind.: Non-induced; ind.: Induced; ASC_ischemic_: ASCs cultured under ischemic conditions before induction; ASC_control_: ASCs cultured in growth medium before induction.

In addition, we examined whether ASCs were still able to differentiate adipogenically after being subjected to ischemia-like stress conditions. For this purpose, ASCs were cultured under glucose/oxygen deprivation (0.1 g/L glucose, 0.2% O_2_) for four days and subsequently differentiated in adipogenic medium. Control ASCs were cultured under standard conditions (growth medium, 21% O_2_) and subsequently differentiated in the same way. Lipid accumulation was analyzed histologically and by quantification of intracellular lipid content. Similar amounts of stained lipid droplets were observed in ASCs previously treated with glucose/oxygen deprivation and in control ASCs (Figure 2A). This finding was corroborated by the triglyceride assay, which showed comparable amounts of intracellular triglycerides in both groups (Figure 2B). Thus, ASCs were not affected in their adipogenic differentiation capability after exposure to ischemia-mimicking conditions.

**Figure 3:**
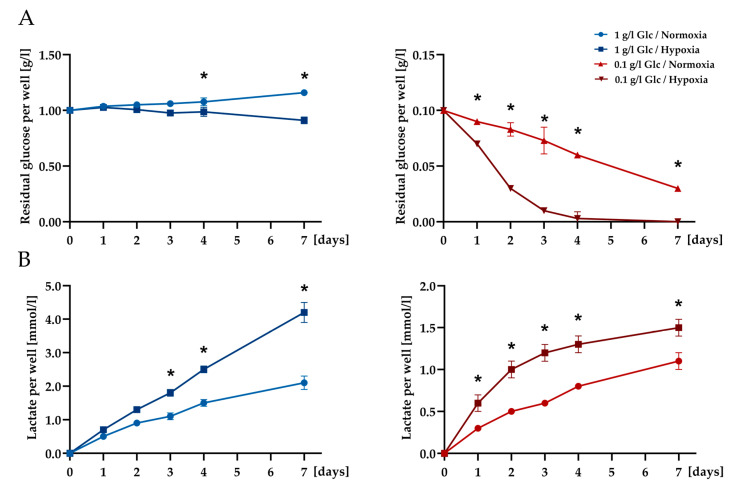
Glucose consumption and lactate production of ASCs cultured with 1 or 0.1 g/L glucose exposed to 21% or 0.2% oxygen over seven days. (**A**) Time course of exogenous glucose levels in the supernatant media (per well). (**B**) Time course of lactate production (per well). Data are presented as means ± SD, *n* = 3; * *p* < 0.05.

To monitor available exogenous glucose levels in the cultures under the different conditions, the remaining glucose concentrations in the culture supernatants were analyzed at different time points. The culture of ASCs in the presence of 0.1 g/L glucose and hypoxia led to a rapid decline in the residual available glucose within the first three days of culture followed by complete exhaustion by day 4. In the cultures supplemented with 1 g/L glucose, the exogenous glucose concentration only slightly decreased in the hypoxic condition as compared to normoxia but was not depleted in either condition over the course of seven days (Figure 3A). Corresponding lactate levels were significantly increased under hypoxic conditions irrespective of the glucose concentration in the media indicating a shift to anaerobic glycolysis under sustained oxygen deprivation (Figure 3B). Under combined glucose/oxygen deprivation, a gradual increase of lactate was observed until day 4 corresponding to the complete exhaustion of glucose, followed by a further slight increase until day 7, which potentially indicates the use of endogenous glycolytic reserves of the cells after complete consumption of the glucose present in the medium.

### 3.2. Secretory Potential of ASCs under Glucose/Oxygen Deprivation

**Figure 4:**
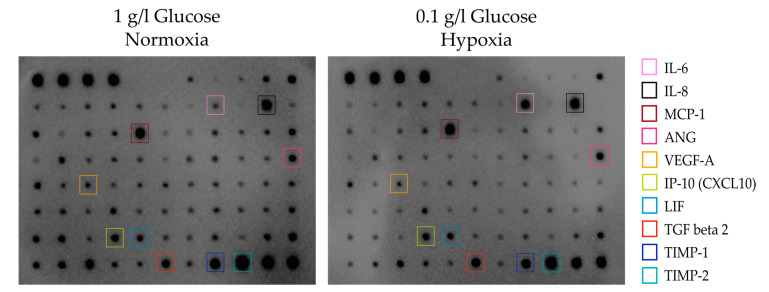
Human cytokine antibody array. Array membranes covering 80 cytokines were probed with supernatants of ASCs collected under control conditions (1 g/L glucose/ 21% O_2_) and glucose/oxygen deprivation (0.1 g/L glucose/ 0.2% O_2_) (*n* = 2). Prominently appearing spots with similar or higher intensity in the deprivation condition were highlighted.

To gain insight into the secretory potential of ASCs exposed to an ischemic environment characterized by nutrient deprivation in combination with acute hypoxic stress, qualitative secretion profiles were established using a cytokine antibody array covering 80 cytokines. To this end, ASCs were cultured under glucose deprivation (0.1 g/L glucose, 21% O_2_) and hypoxia (1 g/L glucose, 0.2% O_2_) and combined glucose/oxygen deprivation (0.1 g/L glucose, 0.2% O_2_). One g/L glucose and 21% O_2_ were set as control condition. Cell culture supernatants were analyzed after four days of culture. It appeared that cells subjected to glucose and oxygen deprivation were able to express a considerable number of growth factors and cytokines to a similar extent or higher as compared to control cells. These factors included angiogenic factors (VEGF, IL-6, IL-8, ANG), matrix-regulating proteins (TIMP-1, TIMP-2), chemokines (MCP-1/CCL2, IP-10/CXCL 10), and others (Figure 4; for comparison, array maps of all four conditions are shown in Figure S2B). Factors that were less pronounced in the glucose/oxygen deprivation condition were also identified, e.g., GRO, FGF-4 and -6, IL-12, IGFBP-2, -3, and -4, NAP-2 (the array map is shown in Figure S2A for identification of the respective spots).

We further quantified the concentration of selected, prominently appearing cytokines (VEGF, IL-6, IL-8, ANG, TIMP-1, MCP-1) from the above panel in the culture supernatants using ELISAs in order to reveal the impact of the individual stress condition (glucose deprivation, hypoxia) on their expression level. STC-1, a peptide hormone, which has been associated with angiogenesis, reduction of apoptosis, and enhanced resistance to metabolic stress [25,26,27], but has so far not been identified as a factor secreted by ASCs, was additionally included in the analysis.

**Figure 5:**
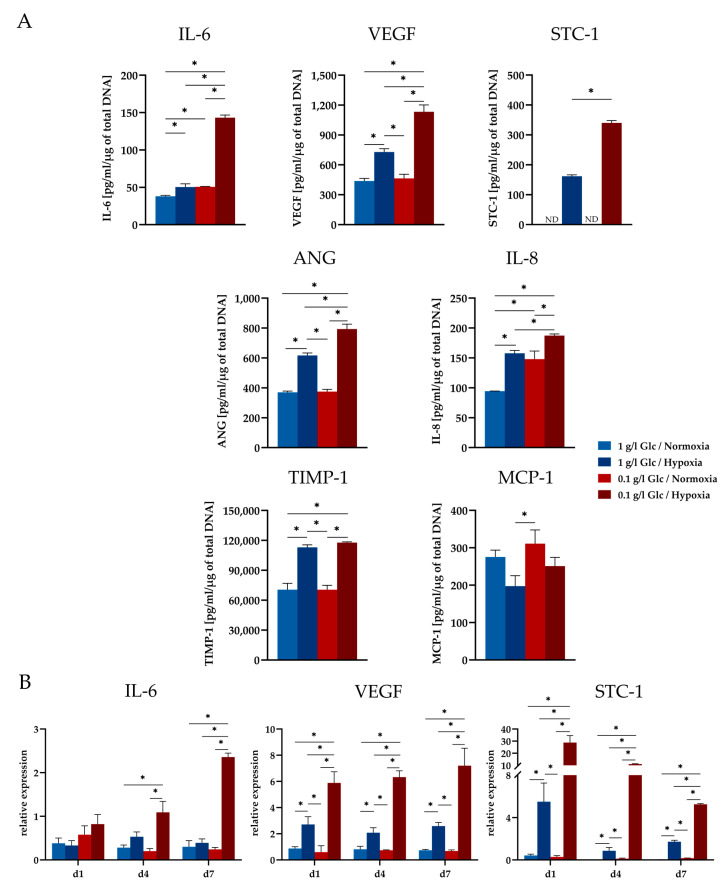
Expression of cytokines in ASCs under deprivation conditions. (**A**) Enzyme-linked immunosorbent assays for IL-6, VEGF, STC-1, ANG, IL-8, TIMP-1, and MCP-1 in the supernatant medium of ASCs cultured under normoxia (21% O_2_) or hypoxia (0.2% O_2_) with either 1 or 0.1 g/L glucose at day 4 of culture; the obtained values were normalized to the DNA content of the respective samples. (**B**) Corresponding gene expression of IL-6, VEGF, and STC-1. Gene expression was normalized to EF1α; the obtained values were further normalized to day 0. (Note: Day 0 values are the same for all groups.) Data are presented as means ± SD of *n* = 3; * *p* < 0.05. Abbreviations: IL-6: Interleukin-6; VEGF: Vascular endothelial growth factor; STC-1: Stanniocalcin-1; ANG: Angiogenin; IL-8: Interleukin-8; TIMP-1: TIMP metallopeptidase inhibitor 1; MCP-1: Monocyte chemoattractant protein-1; ND: Not detectable.

As shown in Figure 5A, increased levels for IL-6, VEGF, STC-1, ANG, IL-8, and TIMP-1 were secreted in response to culture under hypoxic conditions irrespective of the glucose concentration. STC-1 was expressed exclusively under hypoxic conditions. Under normoxic conditions, glucose concentration had no influence on the expression of the selected markers, with the exception of IL-6 and IL-8, which were upregulated under glucose deprivation. Under combined glucose and oxygen deprivation (0.1 g/L glucose, 0.2% O_2_), IL-6, VEGF, and STC-1 levels each showed a striking increase that significantly exceeded the level expressed under hypoxia only (1 g/L glucose, 0.2% O_2_). This effect was also observed for ANG and IL-8 albeit to a lesser extent. TIMP-1 secretion was not affected by the glucose concentration in the culture medium but was increased by hypoxia only. MCP-1 levels remained largely unaltered under all deprivation conditions.

The protein expression of the factors positively regulated by glucose deprivation in the presence of hypoxia was also mirrored on mRNA level. A significantly upregulated gene expression was shown for IL-6, VEGF, and STC-1 under this condition compared to all other conditions. Moreover, different time courses in the expression of these markers were discernible over a period of seven days. STC-1 showed an immediate upregulation upon culture under deprivation conditions with the highest expression level on day 1, whereas IL-6 expression reached its maximum on day 7 of culture. VEGF expression stayed at a constant level over the time course of seven days in this set-up (Figure 5B).

**Figure 6:**
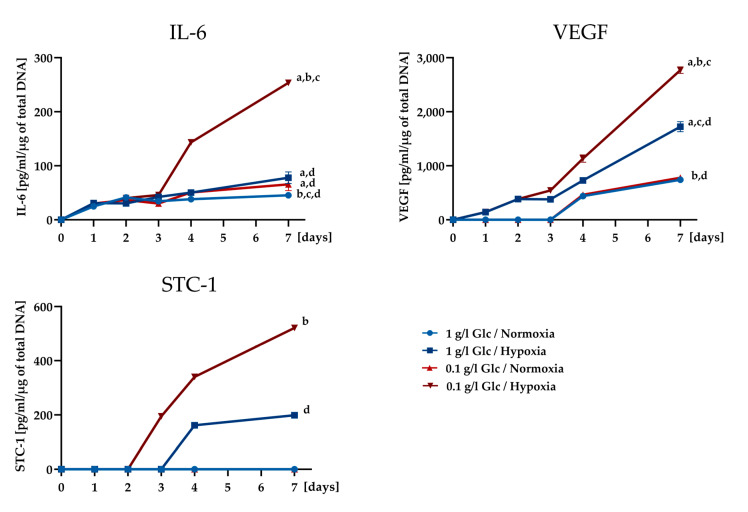
Time course of secretion of IL-6, VEGF, and STC-1 monitored over seven days under different deprivation conditions and quantified by ELISA; the obtained values were normalized to the DNA content of the respective samples. Data are presented as means ± SD of *n* = 3; (**a**) significantly different to the value of group 1 g/L glucose and normoxia at day 7, (**b**) significantly different to the value of group 1 g/L glucose and hypoxia at day 7, (**c**) significantly different to the value of group 0.1 g/L glucose and normoxia at day 7, (**d**) significantly different to the value of group 0.1 g/L glucose and hypoxia at day 7; * *p* < 0.05; for clarity, significance is only shown for day 7. Abbreviations: IL-6: Interleukin-6; VEGF: Vascular endothelial growth factor; STC-1: Stanniocalcin-1; ELISA: Enzyme-linked immunosorbent assay.

In order to gain a deeper insight into how the expression of cytokines depends on the availability of glucose under hypoxia, protein expression levels of IL-6, VEGF, and STC-1 were analyzed in the early phase (until day 4) of culture under deprivation conditions at shorter time intervals. In addition, a later time point (day 7) was included in the analysis to monitor the secretory activity of cells that were completely deprived of glucose. IL-6 showed markedly increased secretion levels under glucose/oxygen deprivation from day 4, when glucose was exhausted in the culture medium (Figure 6). Similarly, an enhanced secretion from day 4 was also evident for VEGF in cultures under combined glucose/oxygen deprivation. STC-1 expression was upregulated under glucose/oxygen deprivation on day 3 and demonstrated a further substantial increase until day 7. The expression of the respective factors remained at significantly lower levels under all other conditions.

These data revealed that ASCs were able to maintain their secretory capacity even under severe glucose/oxygen deprivation over a time course of seven days. Glucose deprivation associated with hypoxia was shown to be an important factor influencing the secretion of the pro-angiogenic and anti-apoptotic factors IL-6, VEGF, and STC-1.

### 3.3. Regenerative Effects of Conditioned Medium from Glucose/Oxygen-Deprived ASCs

**Figure 7:**
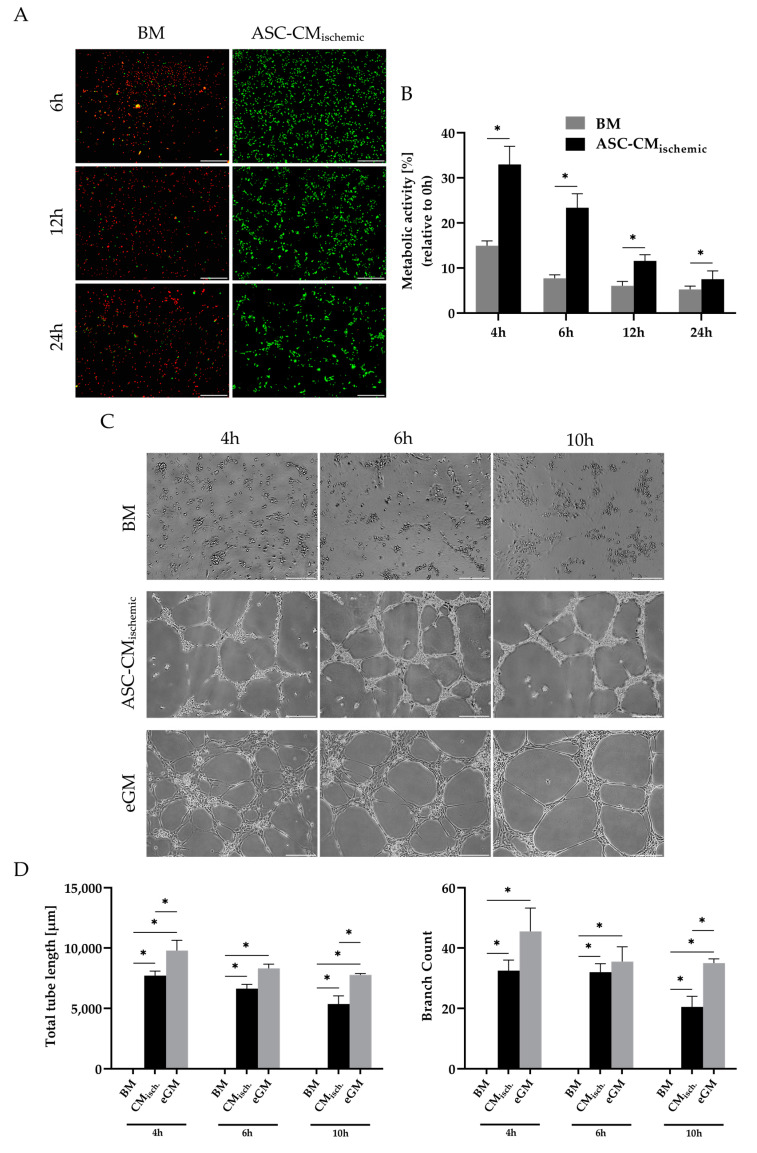
Effect of conditioned medium of glucose/oxygen-deprived ASCs (CM_ischemic_) on viability and angiogenesis of human umbilical vein endothelial cells (HUVECs). (**A**) Live/dead staining at different time points. Viable cells were stained with calcein (green) and dead cells were stained with ethidium bromide (red) at different time points. Scale bar represents 500 μm. (**B**) Determination of metabolic activity by a MTT assay; % metabolic activity was calculated in relation to the mean value of day 0. (**C**) Tube formation by HUVECs in basal medium, ASC-CM_ischemic_ and endothelial growth medium on growth factor-reduced matrigel. Representative micrographs were shown at different time points (4, 6, and 10 h) for illustration of tube formation. Scale bar represents 200 μm. (**D**) Total tube length (left column) and the number of branch points (right column) determined by an automated image analyzer. Data are presented as means ± SD of *n* = 3; * *p* < 0.05. Abbreviations: BM: Basal medium; eGM: Endothelial growth medium; ASC-CM_ischemic_: Adipose-derived stem cell-conditioned medium of glucose/oxygen-deprived ASCs.

We next sought to study the angiogenic properties of conditioned medium from glucose/oxygen-deprived ASCs as this condition corresponds to the ischemic situation at the transplantation site. To this end, we cultured human umbilical vein endothelial cells (HUVECs) in basal medium conditioned by ASCs under glucose/oxygen deprivation and compared them to HUVECs cultured in basal medium. Viability, metabolic activity, and tube formation were assessed. Live/dead staining revealed a distinctly better viability of HUVECs in the presence of conditioned medium over a period of 24 h. In comparison, HUVECs in basal medium died rapidly, after 6 h no viable cells could be detected (Figure 7A). The positive impact of the conditioned medium from glucose/oxygen-deprived ASCs was also reflected in the metabolic activity of the endothelial cells. The metabolic activity determined by a MTT assay was significantly higher in the ASC-conditioned medium than in the basal medium (Figure 7B). The pro-angiogenic effect of secreted factors from glucose/oxygen-deprived ASCs was further analyzed by a tube formation assay on growth factor-reduced matrigel. Figure 7C illustrates the distinct formation of tubular networks after incubation with conditioned medium at different time points (4, 6, and 10 h). In the unconditioned basal medium, HUVECs were not able to develop tube-like structures. The tube formation in endothelial growth medium is shown as a positive control. Accordingly, the tube length and the number of branch points were significantly higher at all time points in the conditioned medium group as compared to the basal medium-treated group (Figure 7D). These results indicated that ASCs exposed to ischemia-mimicking conditions were able to restore endothelial cell viability, metabolic activity, and tube formation via their trophic function.

**Figure 8:**
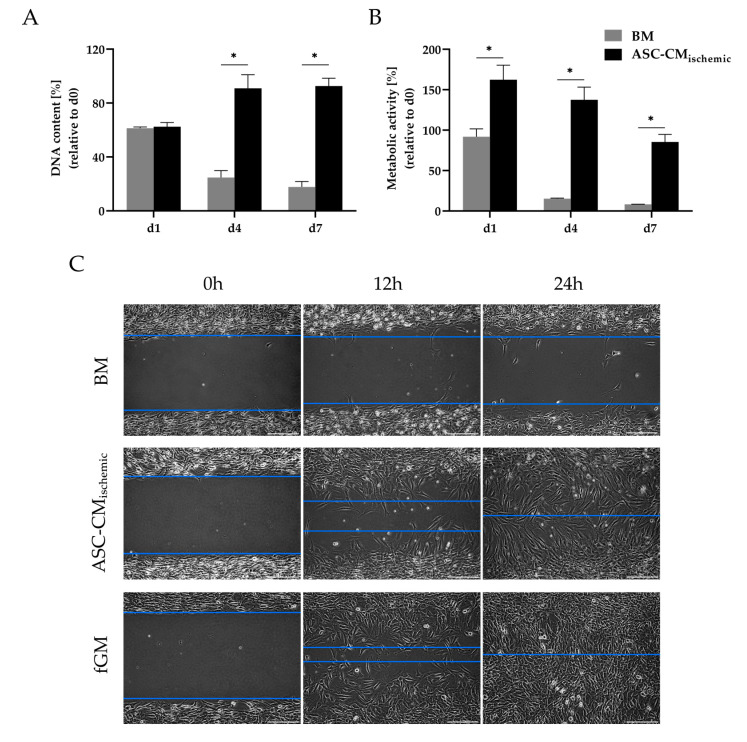
Effect of conditioned medium of glucose/oxygen-deprived ASCs (CM_ischemic_) on proliferation, metabolic activity, and migration capacity of NIH/3T3 fibroblasts. (**A**) Quantitative determination of total DNA content in relation to the mean value of day 0. (**B**) Metabolic activity as determined by a MTT assay. MTT accumulated in fibroblasts was solubilized and the optical density was measured at 570 nm; % metabolic activity was calculated in relation to the mean value of day 0. Data are presented as means ± SD of *n* = 3; * *p* < 0.05. (**C**) Migration of fibroblasts in conditioned medium from glucose/oxygen-deprived ACSs in comparison to the basal medium. Fibroblast growth medium served as a positive control. Representative micrographs at 0, 12, and 24 h were chosen for illustration, blue lines indicate the migration front. Scale bar represents 200 µm. Data are presented as means ± SD of *n* = 3; * *p* < 0.05. Abbreviation: MTT: 3-(4,5-dimethylthiazol-2-yl)-2,5-diphenyltetrazolium bromide; BM: Basal medium, ASC-CM_ischemic_: Adipose-derived stem cell-conditioned medium of glucose/oxygen-deprived ASCs; fGM: Fibroblast growth medium.

In addition, the effect of the secreted factors from ischemia-challenged ASCs on the proliferation and migration of fibroblasts was investigated, since the activation of fibroblasts, in addition to angiogenesis, is considered as an important factor in wound healing processes. In the presence of conditioned medium from glucose/oxygen-deprived ASCs, the proliferation (reflected by the DNA content, Figure 8A) and metabolic activity (as assessed by the MTT assay, Figure 8B) of fibroblasts was markedly enhanced over a period of seven days. The migration assay further illustrates a clear stimulating effect of conditioned medium from glucose/oxygen-deprived ASCs on the migratory activity of fibroblasts compared to the basal medium. The migration of fibroblasts in fibroblast growth medium is shown as a positive control (Figure 8C). Thus, ASCs in an ischemic environment might be able to exert positive effects on wound healing processes through the promotion of neo-vascularization and the activation of fibroblasts in terms of proliferation and migration. In contrast, the growth rate and metabolic activity of breast cancer cell lines (MCF-7 and MDA-MB-231) were not significantly increased by the trophic factors of ASCs under glucose/oxygen deprivation (except for the metabolic activity in MDA-MB-231 on day 1) (Figure S3). This may be relevant in cell-assisted lipotransfer after mastectomy, a scenario in which ischemic conditions at the transplant site are likely to occur.

## 4. Discussion

The positive effects of ASC-based approaches in regenerative therapies have been demonstrated in preclinical and clinical studies, for example in cell-assisted lipotransfer or treatment of ischemic diseases [9,28,29,30]. However, a substantial loss of implanted cells has been documented during the early phase of engraftment [12,31]. Since ASCs, after transplantation into damaged tissues, are exposed to an ischemic environment characterized by the deprivation of nutrients and oxygen, a better understanding of the mechanisms underlying the beneficial effects in the early phase following transplantation is required. This would contribute to a more rational application of ASCs in cell-based approaches. The regenerative potential of ASCs nowadays is mainly attributed to their trophic activity through the secretion of angiogenic, anti-apoptotic, and immuno-modulatory factors. Hypoxia as one of the hallmarks of ischemia is well reported to enhance the secretion of such factors [32,33,34]. However, little is known about the ability of ASCs to maintain their secretory function under starvation conditions. Thus, we investigated the viability and secretory function of ASCs in response to glucose deprivation and severe hypoxia and the combination of both as major stress conditions in an ischemic environment. To our knowledge, no study has yet been conducted to investigate the effect of glucose deprivation as one of the components of ischemia on the secretion capacity of ASCs.

The hallmarks of ischemia were simulated in our in vitro set-up by culturing the ASCs under glucose deprivation (0.1 g/L glucose) and severe hypoxia (0.2% O_2_) alone and in combination over a period of seven days in serum-free medium to mimic ischemic conditions during the early post-transplantation phase. Serum-free culture in combination with oxygen and also glucose deprivation is a widely used experimental model to mimic ischemic conditions in vitro. Furthermore, serum-free culture is also a common approach in studies analyzing the effects of secreted proteins by using a conditioned medium in order to avoid interference with serum proteins [35]. Viability and cell morphology were shown to be virtually not affected by the applied stress conditions over the culture period of seven days. Sustained viability of ASCs in response to adverse nutrient and oxygen levels was also reported by Mischen et al. [23] in a comparable set-up and time frame. In contrast to the limited cell death, the metabolic activity of the cells was significantly affected by glucose limitation from day 1, whereas severe hypoxia did not particularly influence the metabolic activity. Under the glucose-limiting condition (0.1 g/L), cells faced a complete exhaustion of glucose from day 4 under hypoxia, whereas the glucose level decreased more slowly in the normoxic condition. One g/L glucose was not limiting in this setup. In general, glucose levels in the medium demonstrated a steeper decline of the available glucose, when cells were exposed to hypoxia. The corresponding increase of lactate as an important by-product in glycolysis indicated that ASCs increasingly rely on anaerobic glycolysis for their metabolic demands when exposed to hypoxic conditions. Several studies exploring the metabolism of bone marrow-derived mesenchymal stem cells (MSCs) under glucose and oxygen deprivation demonstrated the metabolic flexibility of MSCs under such adverse conditions and their ability to rely on anaerobic glycolysis for energy supply in a hypoxic environment [21,22,36]. In this context, the crucial role of glucose for MSCs function in a hypoxic environment was emphasized. In the present study, the cells were able to maintain their viability for several days despite the complete exhaustion of glucose. This could possibly be due to an enhanced autophagic activity of the cells, as autophagy has been shown to be a survival mechanism for oxygen/glucose-deprived MSCs [36,37,38]. A further observation was that those ASCs that survived under the harsh ischemic condition (glucose/oxygen deprivation) were not affected in their adipogenic differentiation capability. This finding additionally underlined the remarkable resilience of hASCs to an adverse environment.

The next step was to examine the secretion of the ASCs exposed to oxygen and/or glucose deprivation to determine whether cells under ischemic stress were able to maintain their secretory function. As displayed by a cytokine antibody array, ASCs were able to express a broad range of growth factors, cytokines, and chemokines, which in part appeared to be stimulated by hypoxia. When glucose deprivation was combined with hypoxia in order to mimic ischemia, ASCs were still able to maintain secretory function. Under this condition, the cells expressed growth factors and cytokines with angiogenic (VEGF, IL-6, IL-8, ANG) and matrix-remodeling (TIMP-1, TIMP-2) functions and chemokines (MCP-1/CCL2, IP-10/CXCL10) among others, while cytokines associated for example with the regulation of proliferation, cell division, and differentiation appeared to be expressed to a lesser extent (e.g., FGF-4 and -6, IGFBP-2, -3, and -4, NAP-2).

To reveal the impact of the individual stress condition on the expression level of selected factors (VEGF, IL-6, IL-8, ANG, TIMP-1, and MCP-1), their expression was investigated under glucose and oxygen deprivation separately and in combination. STC-1 was included in the analysis as a factor associated with the reduction of apoptosis, angiogenesis, and enhanced resistance of cells to metabolic stress [25,26,27]. Furthermore, STC-1 appears to be closely related to cellular metabolism, as a role of STC-1 in the activation of AMP-activated protein kinase (AMPK) has been postulated. AMPK in turn is a key regulator in the cellular adaptive response to ischemia [39]. STC-1 was shown to be expressed by different cell types including bone marrow-derived MSCs [25,40,41,42] but is a still unknown factor in ASCs. Thus, to the best of our knowledge, the secretion of STC-1 by ASCs was demonstrated for the first time in this study. We found different response patterns of the investigated factors to the individual stress conditions. It is generally accepted that hypoxia triggers the expression of a variety of growth factors and cytokines in ASCs [32,33,43]. Accordingly, we also found an increase in the expression of most of the factors investigated, when the cells were exposed to 0.2% O_2_ (VEGF, IL-6, IL-8, ANG, TIMP-1, STC-1). MCP-1 showed no response to reduced oxygen levels. However, when hypoxia was combined with glucose deprivation in order to mimic ischemia, the secretion of VEGF, IL-6, IL-8, ANG, and STC-1, which are all factors with angiogenic and/or anti-apoptotic properties, increased markedly compared to the hypoxic condition alone. Thus, glucose deprivation (in conjunction with hypoxia) proved to be a factor that positively influenced the secretion of angiogenic and anti-apoptotic cytokines. The availability of glucose as a variable that influences the secretion performance of cells has hardly been investigated so far. Bakopoulou et al. [44] examined the secretion of human apical papilla mesenchymal stem cells subjected to glucose and oxygen deprivation and they also reported a stimulating effect of glucose deprivation on the secretion of angiogenic growth factors in conjunction with hypoxia. In addition, VEGF has been described in early studies with glioma tumor cells as a “classical stress-induced gene”, whose secretion was enhanced by oxygen and glucose deficiency [45,46]. In contrast, Deschepper et al. [47] considered glucose essential for the response of hMSC to near-anoxic conditions. They reported a moderate increase in VEGF-C secretion with increasing glucose concentrations under severe hypoxia in MSCs. With regard to IL-6, the elevated expression under both glucose and oxygen deprivation determined in the present study is in accordance with reports that glucose deprivation triggers IL-6 expression by activation of ER stress signaling pathways [48,49]. The consideration of the time course of secretion additionally underlined the effect of glucose deficiency on the secretion of ASCs with a distinct increase in the levels of IL-6 and VEGF from the time point of complete glucose exhaustion in the culture (day 4). STC-1 secretion was detectable from day 3 under glucose/oxygen deprivation and was maintained until day 7 with a significant increase as compared to hypoxia alone. Gene expression of STC-1 was immediately upregulated in response to glucose/oxygen deprivation. This may indicate that STC-1 possibly elicits a stimulatory effect on VEGF expression. Several studies have shown that the expression of VEGF is associated with STC-1 and a positive feedback-loop between STC-1 and VEGF stimulation has been postulated [50,51].

Maintaining endothelial function and promoting angiogenesis in an ischemic environment to ensure adequate blood supply are considered key processes in regenerative approaches such as cell-assisted lipotransfer or treatment of ischemic diseases. The angiogenic function of secreted growth factors and cytokines of (co-)implanted ASCs could play an important role in this context [30,52]. For this reason, we investigated the impact of the secretome of ischemia-challenged ASCs on the viability, metabolic activity, and tube formation of endothelial cells (HUVECs). The results indicated that ASCs exposed to ischemia-mimicking conditions were able to restore endothelial cell viability, metabolic activity, and tube formation via their secretory function. The angiogenic response to VEGF, IL-6, IL-8, and ANG, which were shown to be major factors secreted under this condition, has been well documented [53,54,55,56,57]. Further studies are necessary to clarify to what extent the newly detected STC-1 in ASCs may contribute to this effect.

ASCs have further been shown to improve wound healing by promoting angiogenesis and fibroblast activation through their paracrine action [58,59,60]. Here, we demonstrated that factors secreted by ASCs in an ischemia-like environment, besides their angiogenic activity, stimulated the proliferation, metabolic activity, and migration of fibroblasts. IL-6 and IL-8 are known as factors that play an important role in wound healing by triggering fibroblast and keratinocyte migration, leukocyte infiltration, and collagen synthesis [61,62,63]. MCP-1 (CCL-2), which was also prominently expressed by oxygen/glucose-deprived ASCs, is a further important chemokine involved in wound healing processes, acting mainly through the recruitment of macrophages [64]. Altogether, the findings support the regenerative potential of ischemia-challenged ASCs in wound healing, for example in chronic ischemic wounds. In contrast, the proliferation of breast cancer cell lines (MCF-7 and MDA-MB-231) was not increased in the presence of trophic factors from ASCs cultured under ischemia-like stress conditions. This may be relevant in the context of cell-assisted lipografting after mastectomy, where ischemic conditions at the transplantation site are likely to prevail. This observation may also be in line with reports from clinical studies that have suggested no increase in cancer recurrence rates in breast cancer patients treated with ASC-enriched lipografts, but further studies would be needed to more specifically assess the effects of the secretome of ischemia-challenged ASCs on breast cancer cells [65,66].

## 5. Conclusions

In conclusion, the present study demonstrated that ASCs showed sustained viability and metabolic flexibility under glucose deprivation and severe hypoxia as hallmarks of ischemia and were able to maintain their secretory function. The secretion of angiogenic and anti-apoptotic factors was influenced not only by hypoxia but was distinctly increased in conjunction with glucose deprivation. Under this condition, the ASCs also expressed STC-1, a factor with angiogenic and anti-apoptotic properties, but which is as yet unknown in ASCs. These results provide valuable insights into how ASCs, through their robustness and remarkable secretory function, may mediate regenerative effects even under adverse conditions such as those found at implantation sites or in damaged tissue.

## Supplementary Materials

The following are available online at https://www.mdpi.com/2073-4409/9/9/1935/s1. Figure S1: Metabolic activity of human ASCs under glucose and oxygen deprivation, determined by the WST-8 assay; Figure S2: Human cytokine antibody array. (A) The map of the array (RayBio^®^ human cytokine array C5, RayBiotech, Norcross, GA) showing the position of 80 human cytokines and positive and negative controls (as available on https://doc.raybiotech.com/pdf/Manual/AAH-CYT-5.pdf). (B) Array membranes probed with supernatants of ASCs collected under 1 g/L glucose and normoxia (21% O_2_) (control), 1 g/L glucose and hypoxia (0.2% O_2_), 0.1 g/L glucose and normoxia (21% O_2_), and 0.1 g/L glucose and hypoxia (0.2% O_2_) at day 4; Figure S3: Effect of conditioned medium of glucose/oxygen-deprived ASCs (CM_ischemic_) on growth rate and metabolic activity of MCF-7 and MDA-MB-231 breast cancer cell lines. The Supporting Material contains additional information on the WST-8 assay and on culture conditions of breast cancer cells (MCF-7 and MDA-MDA-231).

## References

[1] Si Z., Wang X., Sun C., Kang Y., Xu J., Wang X., Hui Y. (2019). Adipose-derived stem cells: Sources, potency, and implications for regenerative therapies. Biomed. Pharmacother..

[2] Gimble J.M., Katz A.J., Bunnell B.A. (2007). Adipose-derived stem cells for regenerative medicine. Circ. Res..

[3] Dai R., Wang Z., Samanipour R., Koo K.I., Kim K. (2016). Adipose-Derived Stem Cells for Tissue Engineering and Regenerative Medicine Applications. Stem Cells Int..

[4] Choi J.H., Gimble J.M., Lee K., Marra K.G., Rubin J.P., Yoo J.J., Vunjak-Novakovic G., Kaplan D.L. (2010). Adipose tissue engineering for soft tissue regeneration. Tissue Eng. Part B Rev..

[5] Kim W.S., Han J., Hwang S.J., Sung J.H. (2014). An update on niche composition, signaling and functional regulation of the adipose-derived stem cells. Expert Opin. Biol. Ther..

[6] Bauer-Kreisel P., Goepferich A., Blunk T. (2010). Cell-delivery therapeutics for adipose tissue regeneration. Adv. Drug Deliv. Rev..

[7] Yoshimura K., Sato K., Aoi N., Kurita M., Hirohi T., Harii K. (2008). Cell-assisted lipotransfer for cosmetic breast augmentation: Supportive use of adipose-derived stem/stromal cells. Aesthetic Plast. Surg..

[8] Vyas K.S., Vasconez H.C., Morrison S., Mogni B., Linton S., Hockensmith L., Kabir T., Zielins E., Najor A., Bakri K. (2020). Fat Graft Enrichment Strategies: A Systematic Review. Plast. Reconstr. Surg..

[9] Kølle S.F.T., Fischer-Nielsen A., Mathiasen A.B., Elberg J.J., Oliveri R.S., Glovinski P.V., Kastrup J., Kirchhoff M., Rasmussen B.S., Talman M.L.M. (2013). Enrichment of autologous fat grafts with ex-vivo expanded adipose tissue-derived stem cells for graft survival: A randomised placebo-controlled trial. Lancet.

[10] Salgado A.J., Reis R.L., Sousa N., Gimble J.M. (2010). Adipose Tissue Derived Stem Cells Secretome: Soluble Factors and Their Roles in Regenerative Medicine. Curr. Stem Cell Res. Ther..

[11] Li X., Ma T., Sun J., Shen M., Xue X., Chen Y., Zhang Z. (2019). Harnessing the secretome of adipose-derived stem cells in the treatment of ischemic heart diseases. Stem Cell Res. Ther..

[12] Mooney D.J., Vandenburgh H. (2008). Cell Delivery Mechanisms for Tissue Repair. Cell Stem Cell.

[13] Chen L., Tredget E.E., Wu P.Y.G., Wu Y.Y.G. (2008). Paracrine Factors of Mesenchymal Stem Cells Recruit Macrophages and Endothelial Lineage Cells and Enhance Wound Healing. PLoS ONE.

[14] Ferreira J.R., Teixeira G.Q., Santos S.G., Barbosa M.A., Almeida-Porada G., Gonçalves R.M. (2018). Mesenchymal Stromal Cell Secretome: Influencing Therapeutic Potential by Cellular Pre-conditioning. Front. Immunol..

[15] Praveen Kumar L., Sangeetha K., Ranjita M., Vijayalakshmi S., Rajagopal K., Rama Shanker V. (2019). The mesenchymal stem cell secretome: A new paradigm towards cell-free therapeutic mode in regenerative medicine. Cytokine Growth Factor Rev..

[16] Meiliana A., Dewi N.M., Wijaya A. (2019). Mesenchymal stem cell secretome: Cell-free therapeutic strategy in regenerative medicine. Indones. Biomed. J..

[17] Fu Y., Karbaat L., Wu L., Leijten J., Both S.K., Karperien M. (2017). Trophic Effects of Mesenchymal Stem Cells in Tissue Regeneration. Tissue Eng. Part B Rev..

[18] Weiser B., Prantl L., Schubert T.E.O., Zellner J., Fischbach-Teschl C., Spruss T., Seitz A.K., Tessmar J., Goepferich A., Blunk T. (2008). In Vivo Development and Long-Term Survival of Engineered Adipose Tissue Depend on In Vitro Precultivation Strategy. Tissue Eng. Part A.

[19] Lu F., Li J., Gao J., Ogawa R., Ou C., Yang B., Fu B. (2009). Improvement of the survival of human autologous fat transplantation by using VEGF-transfected adipose-derived stem cells. Plast. Reconstr. Surg..

[20] Moya A., Paquet J., Deschepper M., Larochette N., Oudina K., Denoeud C., Bensidhoum M., Logeart-Avramoglou D., Petite H. (2018). Human Mesenchymal Stem Cell Failure to Adapt to Glucose Shortage and Rapidly Use Intracellular Energy Reserves Through Glycolysis Explains Poor Cell Survival After Implantation. Stem Cells.

[21] Deschepper M., Oudina K., David B., Myrtil V., Collet C., Bensidhoum M., Logeart-Avramoglou D., Petite H. (2011). Survival and function of mesenchymal stem cells (MSCs) depend on glucose to overcome exposure to long-term, severe and continuous hypoxia. J. Cell. Mol. Med..

[22] Mylotte L.A., Duffy A.M., Murphy M., O’Brien T., Samali A., Barry F., Szegezdi E. (2008). Metabolic Flexibility Permits Mesenchymal Stem Cell Survival in an Ischemic Environment. Stem Cells.

[23] Mischen B.T., Follmar K.E., Moyer K.E., Buehrer B., Olbrich K.C., Levin L.S., Klitzman B., Erdmann D. (2008). Metabolic and functional characterization of human adipose-derived stem cells in tissue engineering. Plast. Reconstr. Surg..

[24] Faghih H., Javeri A., Taha M.F. (2017). Impact of early subcultures on stemness, migration and angiogenic potential of adipose tissue-derived stem cells and their resistance to in vitro ischemic condition. Cytotechnology.

[25] Block G.J., Ohkouchi S., Fung F., Frenkel J., Gregory C., Pochampally R., Dimattia G., Sullivan D.E., Prockop D.J. (2009). Multipotent Stromal Cells (MSCs) are Activated to Reduce Apoptosis in Part by Upregulation and Secretion of Stanniocalcin-1 (STC-1) HHS Public Access. Stem Cells.

[26] Yeung B.H.Y., Law A.Y.S., Wong C.K.C. (2012). Evolution and roles of stanniocalcin. Mol. Cell. Endocrinol..

[27] Chen F., Zhang Z., Pu F. (2019). Role of stanniocalcin-1 in breast cancer (Review). Oncol. Lett..

[28] He X., Zhong X., Ni Y., Liu M., Liu S., Lan X. (2013). Effect of ASCs on the graft survival rates of fat particles in rabbits. J. Plast. Surg. Hand Surg..

[29] Matsumoto D., Sato K., Gonda K., Takaki Y., Shigeura T., Sato T., Aiba-Kojima E., Iizuka F., Inoue K., Suga H. (2006). Cell-assisted lipotransfer: Supportive use of human adipose-derived cells for soft tissue augmentation with lipoinjection. Tissue Eng..

[30] Zhao L., Johnson T., Liu D. (2017). Therapeutic angiogenesis of adipose-derived stem cells for ischemic diseases. Stem Cell Res. Ther..

[31] Copland I.B., Lord-Dufour S., Cuerquis J., Coutu D.L., Annabi B., Wang E., Galipeau J. (2009). Improved Autograft Survival of Mesenchymal Stromal Cells by Plasminogen Activator Inhibitor 1 Inhibition. Stem Cells.

[32] Rehman J., Traktuev D., Li J., Merfeld-Clauss S., Temm-Grove C.J., Bovenkerk J.E., Pell C.L., Johnstone B.H., Considine R.V., March K.L. (2004). Secretion of Angiogenic and Antiapoptotic Factors by Human Adipose Stromal Cells. Circulation.

[33] Frazier T.P., Gimble J.M., Kheterpal I., Rowan B.G. (2013). Impact of low oxygen on the secretome of human adipose-derived stromal/stem cell primary cultures. Biochimie.

[34] Madrigal M., Rao K.S., Riordan N.H. (2014). A review of therapeutic effects of mesenchymal stem cell secretions and induction of secretory modification by different culture methods. J. Transl. Med..

[35] Pirkmajer S., Chibalin A.V. (2011). Serum starvation: Caveat emptor. Am. J. Physiol. Cell Physiol..

[36] Nuschke A., Rodrigues M., Wells A.W., Sylakowski K., Wells A. (2016). Mesenchymal stem cells/multipotent stromal cells (MSCs) are glycolytic and thus glucose is a limiting factor of in vitro models of MSC starvation. Stem Cell Res. Ther..

[37] Moya A., Larochette N., Paquet J., Deschepper M., Bensidhoum M., Izzo V., Kroemer G., Petite H., Logeart-Avramoglou D. (2017). Quiescence Preconditioned Human Multipotent Stromal Cells Adopt a Metabolic Profile Favorable for Enhanced Survival under Ischemia. Stem Cells.

[38] Li C., Ye L., Yang L., Yu X., He Y., Chen Z., Li L., Zhang D. (2018). Rapamycin Promotes the Survival and Adipogenesis of Ischemia-Challenged Adipose Derived Stem Cells by Improving Autophagy. Cell. Physiol. Biochem..

[39] Pan J.S.C., Huang L., Belousova T., Lu L., Yang Y., Reddel R., Chang A., Ju H., DiMattia G., Tong Q. (2015). Stanniocalcin-1 inhibits renal ischemia/reperfusion injury via an AMP-activated protein kinase-dependent pathway. J. Am. Soc. Nephrol..

[40] Higuera G.A., Fernandes H., Spitters T.W.G.M., van de Peppel J., Aufferman N., Truckenmueller R., Escalante M., Stoop R., van Leeuwen J.P., de Boer J. (2015). Spatiotemporal proliferation of human stromal cells adjusts to nutrient availability and leads to stanniocalcin-1 expression in vitro and in vivo. Biomaterials.

[41] Bironaite D., Westberg J.A., Andersson L.C., Venalis A. (2013). A variety of mild stresses upregulate stanniocalcin-1 (STC-1) and induce mitohormesis in neural crest-derived cells. J. Neurol. Sci..

[42] Westberg J.A., Serlachius M., Lankila P., Penkowa M., Hidalgo J., Andersson L.C. (2007). Hypoxic preconditioning induces neuroprotective stanniocalcin-1 in brain via IL-6 signaling. Stroke.

[43] Paquet J., Deschepper M., Moya A., Logeart-Avramoglou D., Boisson-Vidal C., Petite H. (2015). Oxygen Tension Regulates Human Mesenchymal Stem Cell Paracrine Functions. Stem Cells Transl. Med..

[44] Bakopoulou A., Kritis A., Andreadis D., Papachristou E., Leyhausen G., Koidis P., Geurtsen W., Tsiftsoglou A. (2015). Angiogenic Potential and Secretome of Human Apical Papilla Mesenchymal Stem Cells in Various Stress Microenvironments. Stem Cells Dev..

[45] Stein I., Neeman M., Shweiki D., Itin A., Keshet E. (1995). Stabilization of vascular endothelial growth factor mRNA by hypoxia and hypoglycemia and coregulation with other ischemia-induced genes. Mol. Cell. Biol..

[46] Shweiki D., Neeman M., Itin A., Keshet E. (1995). Induction of vascular endothelial growth factor expression by hypoxia and by glucose deficiency in multicell spheroids: Implications for tumor angiogenesis. Proc. Natl. Acad. Sci. USA.

[47] Deschepper M., Manassero M., Oudina K., Paquet J., Monfoulet L.E., Bensidhoum M., Logeart-Avramoglou D., Petite H. (2013). Proangiogenic and prosurvival functions of glucose in human mesenchymal stem cells upon transplantation. Stem Cells.

[48] Choi S.J., Shin I.J., Je K.H., Min E.K., Kim E.J., Kim H.S., Choe S., Kim D.E., Lee D.K. (2013). Hypoxia Antagonizes Glucose Deprivation on Interleukin 6 Expression in an Akt Dependent, but HIF-1/2α Independent Manner. PLoS ONE.

[49] Fougeray S., Bouvier N., Beaune P., Legendre C., Anglicheau D., Thervet E., Pallet N. (2011). Metabolic stress promotes renal tubular inflammation by triggering the unfolded protein response. Cell Death Dis..

[50] He L.F., Wang T.T., Gao Q.Y., Zhao G.F., Huang Y.H., Yu L.K., Hou Y.Y. (2011). Stanniocalcin-1 promotes tumor angiogenesis through up-regulation of VEGF in gastric cancer cells. J. Biomed. Sci..

[51] Law A.Y.S., Wong C.K.C. (2013). Stanniocalcin-1 and -2 promote angiogenic sprouting in HUVECs via VEGF/VEGFR2 and angiopoietin signaling pathways. Mol. Cell. Endocrinol..

[52] Suga H., Glotzbach J.P., Sorkin M., Longaker M.T., Gurtner G.C. (2014). Paracrine mechanism of angiogenesis in adipose-derived stem cell transplantation. Ann. Plast. Surg..

[53] Ferrara N. (1999). Role of vascular endothelial growth factor in the regulation of angiogenesis. Kidney Int..

[54] Nakagami H., Maeda K., Morishita R., Iguchi S., Nishikawa T., Takami Y., Kikuchi Y., Saito Y., Tamai K., Ogihara T. (2005). Novel autologous cell therapy in ischemic limb disease through growth factor secretion by cultured adipose tissue-derived stromal cells. Arterioscler. Thromb. Vasc. Biol..

[55] Karaman S., Leppänen V.M., Alitalo K. (2018). Vascular endothelial growth factor signaling in development and disease. Development.

[56] Pu C.M., Liu C.W., Liang C.J., Yen Y.H., Chen S.H., Jiang-Shieh Y.F., Chien C.L., Chen Y.C., Chen Y.L. (2017). Adipose-Derived Stem Cells Protect Skin Flaps against Ischemia/Reperfusion Injury via IL-6 Expression. J. Investig. Dermatol..

[57] Fan Y., Ye J., Shen F., Zhu Y., Yeghiazarians Y., Zhu W., Chen Y., Lawton M.T., Young W.L., Yang G.Y. (2008). Interleukin-6 stimulates circulating blood-derived endothelial progenitor cell angiogenesis in vitro. J. Cereb. Blood Flow Metab..

[58] Hassanshahi A., Hassanshahi M., Khabbazi S., Hosseini-Khah Z., Peymanfar Y., Ghalamkari S., Su Y., Xian C.J. (2019). Adipose-derived stem cells for wound healing. J. Cell. Physiol..

[59] Zhao J., Hu L., Liu J., Gong N., Chen L. (2013). The effects of cytokines in adipose stem cell-conditioned medium on the migration and proliferation of skin fibroblasts in vitro. Biomed Res. Int..

[60] Lee S.H., Jin S.Y., Song J.S., Seo K.K., Cho K.H. (2012). Paracrine effects of adipose-derived stem cells on keratinocytes and dermal fibroblasts. Ann. Dermatol..

[61] Luckett L.R., Gallucci R.M. (2007). Interleukin-6 (IL-6) modulates migration and matrix metalloproteinase function in dermal fibroblasts from IL-6KO mice. Br. J. Dermatol..

[62] Lin Z.-Q., Kondo T., Ishida Y., Takayasu T., Mukaida N. (2003). Essential involvement of IL-6 in the skin wound-healing process as evidenced by delayed wound healing in IL-6-deficient mice. J. Leukoc. Biol..

[63] Michel G., Kemény L., Peter R.U., Beetz A., Ried C., Arenberger P., Ruzicka T. (1992). Interleukin-8 receptor-mediated chemotaxis of normal human epidermal cells. FEBS Lett..

[64] DiPietro L.A., Reintjes M.G., Low Q.E.H., Levi B., Gamelli R.L. (2001). Modulation of macrophage recruitment into wounds by monocyte chemoattractant protein-1. Wound Repair Regen..

[65] Mazur S., Zołocińska A., Siennicka K., Janik-Kosacka K., Chrapusta A., Pojda Z. (2018). Safety of adipose-derived cell (stromal vascular fraction—SVF) augmentation for surgical breast reconstruction in cancer patients. Adv. Clin. Exp. Med..

[66] Waked K., Colle J., Doornaert M., Cocquyt V., Blondeel P. (2017). Systematic review: The oncological safety of adipose fat transfer after breast cancer surgery. Breast.

