# Supplementary material for: Ischemia-Like Stress Conditions Stimulate Trophic Activities of Adipose-Derived Stromal/Stem Cells
Source: Cells. 2020 Aug 21;9(9):1935. doi: 10.3390/cells9091935 (PMC7566001; doi:10.3390/cells9091935)
Supplement: Supplementary file 1 [file cells-09-01935-s001.pdf]

cells

MDPI

Supplementary Material

Ischemia-Like Stress Conditions Stimulate Trophic Activities of Adipose-Derived Stromal/Stem Cells

Julia Bachmann [1], Elias Ehlert [1], Matthias Becker [2], Christoph Otto [3], Katrin Radeloff [4], Torsten Blunk [1] and Petra Bauer-Kreisel [1,*]

Supplementary Methods

Cells and Cell Culture

MCF-7 and MDA-MB-231 breast cancer cells (ATCC) were cultured in Dulbecco´s Modified Eagle´s Medium/Ham´s F-12 (DMEM/F-12) (Thermo Scientific), supplemented with 1% penicillin/streptomycin (Thermo Scientific), and 10% fetal bovine serum (FBS; Thermo Scientific). When the cells reached 80%-85% confluence, they were detached with 0.25% trypsin-EDTA solution (Thermo Scientific) and passaged.

WST-8 Assay

ASCs were seeded in growth medium in 96-well plates and after attachment of cells, they were cultured according to the respective condition. At the indicated time points, 10 µl Cell Counting Kit-8 (Sigma-Aldrich) containing the water-soluble tetrazolium salt WST-8 (2-(2-methoxy-4-nitrophenyl)-3-(4-nitrophenyl)-5-(2,4-disulfophenyl)-2H-tetrazolium, monosodium salt) was directly added into 100 µl culture medium. Cells were then incubated at 37°C for 4 hours. The respective light absorbance of the samples was recorded using a microplate reader (Infinite M200; Tecan, Crailsheim, Germany) at a wavelength of 450nm. The mean value of day 0 samples was taken as reference and set as 100%.

Proliferation and Metabolic Activity of Breast Cancer Cell Lines

Conditioned medium from glucose/oxygen-deprived ASCs (ASC-$CM_{ischemic}$) was prepared as described. Breast cancer cells (MCF-7 and MDA-MB-231) were treated with basal medium (DMEM, w/o FBS) and ASC-$CM_{ischemic}$, each supplemented with 1 g/l glucose, under normoxic conditions. Proliferation and metabolic activity of the cells were analyzed at the indicated time points using a DNA and MTT assay as described.

Supplementary Figures

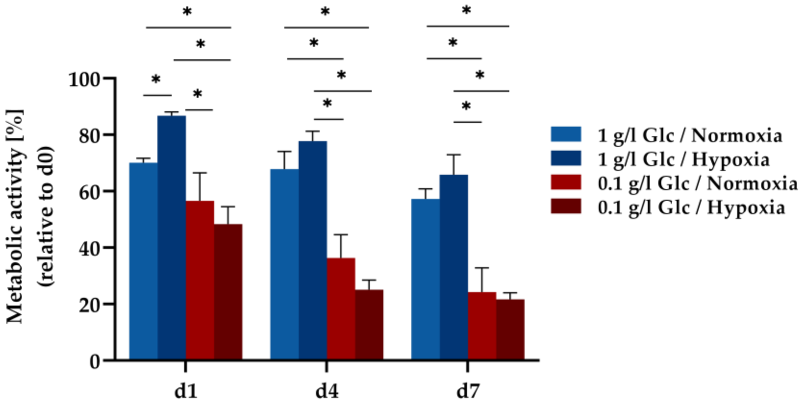

Figure S1. Metabolic activity of human ASCs under glucose and oxygen deprivation, determined by the WST-8 assay. The optical density was measured at 450 nm; % metabolic activity was calculated in relation to the mean value of day 0. Data are presented as means ± SD of n = 3; * $p < .05$. Abbreviation: WST-8, 2-(2-methoxy-4-nitrophenyl)-3- (4-nitrophenyl)-5-(2,4-disulfophenyl)-2H-tetrazolium, monosodium salt.

A

	A	B	C	D	E	F	G	H	I	J	K
1	POS	POS	POS	POS	NEG	NEG	ENA-78 (CXCL5)	G-CSF	GM-CSF	GRO a/b/g	GRO alpha (CXCL1)
2	I-309 (CCL1)	IL-1 alpha (IL-1 F1)	IL-1 beta (IL-1 F2)	IL-2	IL-3	IL-4	IL-5	IL-6	IL-7	IL-8 (CXCL8)	IL-10
3	IL-12 p40/p70	IL-13	IL-15	IFN-gamma	MCP-1 (CCL2)	MCP-2 (CCL8)	MCP-3 (CCL7)	M-CSF	MDC (CCL22)	MIG (CXCL9)	MIP-1 beta (CCL4)
4	MIP-1 delta (CCL15)	RANTES (CCL5)	SCF	SDF-1 alpha	TARC (CCL17)	TGF beta 1	TNF alpha	TNF beta (TNFSF1B)	EGF	IGF-1	Angiogenin
5	OSM	TPO	VEGF-A	PDGF-BB	Leptin	BDNF	BLC (CXCL13)	Ck beta 8-1 (CCL23)	Eotaxin-1 (CCL11)	Eotaxin-2 (CCL24)	Eotaxin-3 (CCL26)
6	FGF-4	FGF-6	FGF-7 (KGF)	FGF-9	FLT-3 Ligand	Fractalkine (CX3CL1)	GCP-2 (CXCL6)	GDNF	HGF	IGFBP-1	IGFBP-2
7	IGFBP-3	IGFBP-4	IL-16	IP-10 (CXCL10)	LIF	LIGHT (TNFSF14)	MCP-4 (CCL13)	MIF	MIP-3 alpha (CCL20)	NAP-2 (CXCL7)	NT-3
8	NT-4	OPN (SPP1)	OPG (TNFRSF11B)	PARC	PLGF	TGF beta 2	TGF beta 3	TIMP-1	TIMP-2	POS	POS

B

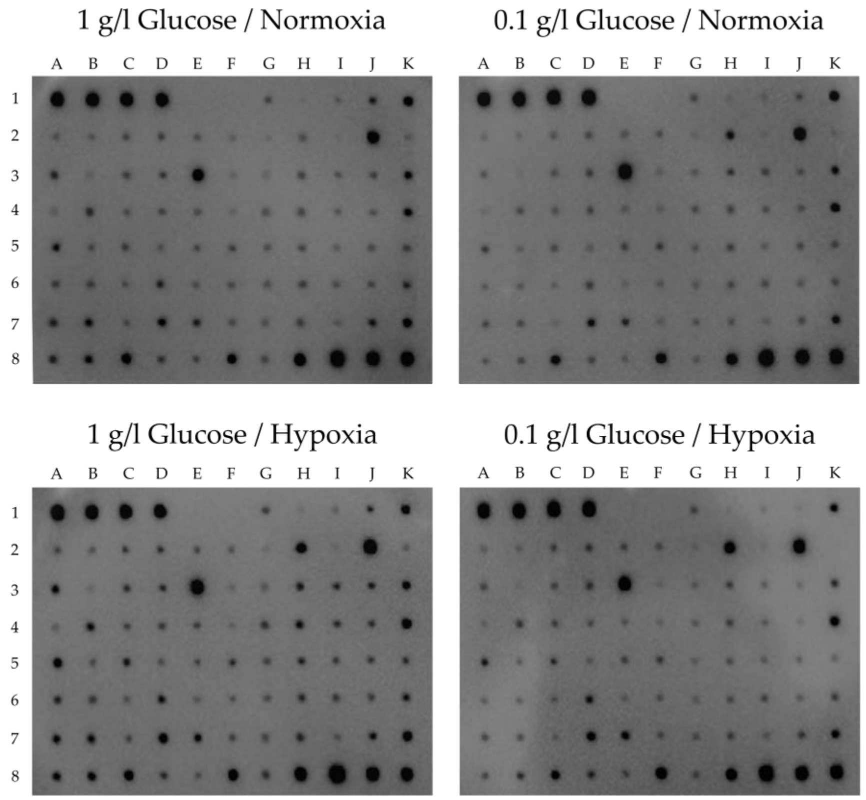

Figure S2. Human cytokine antibody array. (**A**) The map of the array (RayBio® human cytokine array C5, RayBiotech, Norcross, GA) showing the position of 80 human cytokines and positive and negative controls (as available on https://doc.raybiotech.com/pdf/Manual/AAH-CYT-5.pdf). (**B**) Array membranes were probed with supernatants of ASCs collected under 1 g/l glucose and normoxia (21% O_2) (control), 1 g/l glucose and hypoxia (0.2% O_2), 0.1 g/l glucose and normoxia (21% O_2), and 0.1 g/l glucose and hypoxia (0.2% O_2) at day 4 (n=2).

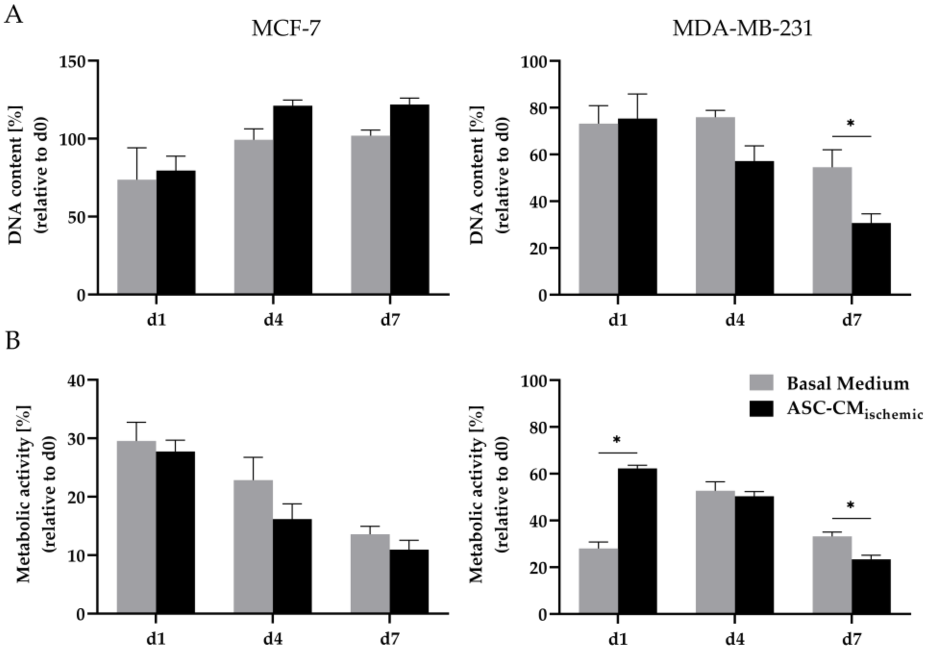

Figure S3. Effect of conditioned medium of glucose/oxygen-deprived ASCs ($CM_{ischemic}$) on MCF-7 and MDA-MB-231 cancer cells growth rate and metabolic activity. (**A**) Quantitative determination of total DNA content in relation to the mean value of day 0. (**B**) Metabolic activity as determined by a MTT assay. MTT accumulated in MCF-7 and MDA-MB-231 was solubilized and the optical density was measured at 570 nm; % metabolic activity was calculated in relation to the mean value of day 0. Data are presented as means ± SD of n = 3; * $p < .05$. Abbreviation: MTT, 3-(4,5-dimethylthiazol-2-yl)- 2,5-diphenyltetrazolium bromide; ASC-$CM_{ischemic}$, adipose-derived stem cell-conditioned medium of glucose/oxygen-deprived ASCs.
